# Cardiovascular involvement in children with COVID-19 temporally related multisystem inflammatory syndrome (MIS-C): can cardiac magnetic resonance arrive to the heart of the problem?

**DOI:** 10.1186/s13052-024-01658-1

**Published:** 2024-05-03

**Authors:** Maria Cristina Maggio, Alessio Lembo, Francesca Finazzo, Annalisa Alaimo, Guglielmo Francesco Benfratello, Giovanni Corsello

**Affiliations:** 1 University Department PROMISE “G. D’Alessandro”, University of Palermo, Via del Vespro 129, 90100 Palermo, Italy; 2 U.O.C. of Paediatric Radiology, Children Hospital “G. Di Cristina”, ARNAS, Palermo, Via dei Benedettini 1, 90100 Palermo, Italy; 3 U.O.C. of Paediatric Cardiac Surgery, ARNAS, Palermo, Via dei Benedettini 1, 90100 Palermo, Italy; 4 University of Pisa, Largo Bruno Pontecorvo 3, 56127 Pisa, Italy

**Keywords:** Multisystem inflammatory syndrome in children (MIS-C), SARS-CoV-2, COVID-19, Cardiac magnetic resonance, Anakinra, Kawasaki disease, Methylprednisolone, Intravenous immunoglobulin

## Abstract

**Background:**

Multisystem inflammatory syndrome in children (MIS-C) shows a significant overlap of symptoms with other hyper-inflammatory diseases such as Kawasaki disease (KD), but the real difference of the two conditions is still matter of debate. Coronary artery lesions (CAL) are the most relevant complication in KD. Nonetheless, CAL, myocarditis, pericarditis, arrhythmia are the main cardiovascular complications in MIS-C. A close clinical assessment is mandatory, both at the diagnosis and during the follow-up, by ECG and echocardiography. Cardiac magnetic resonance (MRI) adds important data to ultrasound findings. However, cardiac MRI studies in MIS-C are limited to a small number of cohorts.

**Methods:**

We enrolled 20 children (age:1–16 years; 11 F; 9 M) with cardiac involvement secondary to MIS-C, all evaluated by cardiac MRI.

**Results:**

8 children showed pathological cardiac MRI: 2 showed pericardial effusion; 2 showed myocardial oedema; 1 showed aortic insufficiency; 3 showed delayed enhancement (one for acute myocarditis with oedema; 2 for myocardial fibrosis). Delayed enhancement was reduced significantly 5.6-9 months after the first MRI evaluation. 25% of patients with pathological MRI had CAL associated with valvular insufficiency of 2 valves. 17% of patients with normal MRI had CAL, associated with valvular insufficiency of 1 valve in 1 patient. The correlations between haematological, clinical, cardiologic parameters, treatment, did not reach the statistical significance. 4 patients were treated with anakinra. Among those, 2 patients showed a normal cardiac MRI. Cardiac lesions resolved in all the patients during the follow-up. Some patients with pathological cardiac MRI could not underwent a control with MRI, for the low compliance. However, echocardiography and ECG, documented the resolution of the pathological data in these cases.

**Conclusions:**

A higher risk of CAL was documented in patients with an association of other cardiac lesions. Cardiac MRI is difficult to perform routinely; however, it is useful for evaluating the acute myocardial damage and the outcome of patients with MIS-C.

**Supplementary Information:**

The online version contains supplementary material available at 10.1186/s13052-024-01658-1.

## Background

MIS-C is a hyperinflammatory syndrome following the exposure to SARS-CoV-2 infection, usually occurring 14–60 days after the SARS-CoV-2 infection, in a patient aged < 21 years.

The Centers for Disease Control and Prevention (CDC) case definition of MIS-C developed a new case definition for MIS-C diagnostic criteria, including: persistent fever > 38 ° C, in the absence of an alternative diagnosis and the exclusion of other microbiological causes, in a clinically severe patient; C-reactive protein (CRP) > 3 mg/dl; > 2 new-onset manifestations of the following: rash, conjunctivitis, oral mucosal changes, erythema and/or oedema of the extremities; vomiting, diarrhoea, abdominal pain; lymphocyte count < 1000/ µl, platelet count < 150,000/µl. The common signs and symptoms include: headache, aseptic meningitis, irritability; respiratory symptoms, myocardial dysfunction, capillary leak and cardiogenic shock; Macrophage Activation Syndrome (MAS), acute kidney injury, thrombosis. increased CRP, erythrocyte sedimentation rate (ESR) and interleukin 6 (IL-6), neutrophilic leukocytosis, lymphopenia and organ dysfunction are secondary to the systemic inflammatory state and/or a cytokine storm [1].

Patients with MIS-C show a significant overlap of symptoms with hyper-inflammatory diseases such as Kawasaki disease (KD) [2–5], but the real difference of the two conditions is still matter of debate.

CAL are the most relevant complication of patients with KD, with different evolution, secondary to the severity of the dilatation, tissue remodelling, prompt diagnosis and therapeutic approach. Most of CAL occur in the proximal segments and in the branch level. However, patients with KD who do not develop CAL at 6 weeks since disease onset, show a good cardiac prognosis. Instead, giant coronary aneurysms are not reversible and CAL are risk factors of coronary arteries thrombosis or/and stenosis [5].

The incidence of CAL varies significantly in patients treated with IVIG and non-treated or who received a late treatment. Furthermore, non-responders need a second step treatment with glucocorticoids and/or anakinra or infliximab [6, 7].

Further cardiovascular complications may occur with a lower incidence in patients with KD, as valvular insufficiency, pericarditis, myocarditis and arrhythmia [5].

Conversely, CAL and/or aneurysms, myocarditis, pericarditis, arrhythmia are the main cardiovascular complications in MIS-C. Hence, a close clinical assessment is mandatory, both at the diagnosis and during the follow-up [8]. As in patients with KD, ECG and echocardiography are the starting point to evaluate myocardial function and coronary artery assessment at the diagnosis and in the acute phase.

The z-score < 2 indicates the absence of coronary artery lesions; the z-score between 2 and 2.5 indicates the presence of coronary artery dilation, and the z-score ≥ 2.5 indicates the presence of coronary artery aneurysm [4, 5].

Cardiac magnetic resonance (MRI) is an accurate diagnostic investigation useful in patients in whom echocardiography is not exhaustive. Cardiac MRI detects myocardial perfusion, myocardial inflammation, biventricular systolic function, oedema, fibrosis, and coronary arteries anatomy and lesions [2, 3].

However, cardiac MRI with gadolinium-based contrast is described in a limited number of studies in children with MIS-C, as its execution often requires general anesthesia in younger children [9–12].

## Methods

This is a retrospective observational, single-center study, performed at the Pediatric Clinic of the Children Hospital “G. Di Cristina”, ARNAS, Palermo, Italy.

We studied a case series of 20 children with cardiac involvement secondary to MIS-C. All the patients fulfilled the ACR case definitions of MIS-C [13], and case definitions of MIS-C are adapted from recommendations from the World Health Organization (WHO) [14] and the Centers for Disease Control and Prevention (CDC) [15] for MIS‐C, as well as the Royal College of Paediatrics and Child Health (RCPCH) for pediatric inflammatory multisystem syndrome temporally associated with SARS–Cov‐2 [1], including any of the following:

- positive SARS-CoV-2 RT-PCR.
- positive serology.
- positive antigen test.
- contact with an individual with COVID-19.
- temperature ≥ 38.0 °C for ≥ 24 h *or* subjective fever for ≥ 24 h.
- severe illness (hospitalized) and ≥ 2 organ or systems involved.
- Laboratory evidence of inflammation including, but not limited to, 1 or more of the following: increased CRP; ESR; fibrinogen; procalcitonin; d-dimer; ferritin; LDH; IL‐6; neutrophilia; lymphopenia; hypoalbuminemia.

All the 20 children included in the study (age: 1–16 years; 11 females; 9 males) (Table 1) were followed by ECG and echocardiography. Additionally, we studied all the patients by cardiac magnetic resonance (MRI) to evaluate heart involvement in children affected by MIS-C.

**Table 1:** Clinical parameters of the patients

Patients (20)	Males	Females	Cardiac follow-up (months)
Gender	9	11	
Race	7 Caucasian; 1 Moroccan; 1 asiatic;	11 Caucasian	
Age (years)	2–16	2–14	
Pathological cardiac MRI	4	4	24
Normal cardiac MRI	5	7	12

All the patients were treated within 72 h after the admission, with IVIG (2 g/Kg), methylprednisolone at the dosage of 2 mg/Kg/day in 100% of patients. 6/20 (30% of patients) were treated with 3 boluses of 30 mg/kg/day of methylprednisolone, followed by 2 mg/Kg/day, due to the severity of the clinical picture and the rapid worsening of the pathology.

Methylprednisolone was gradually tapered, with the end of glucocorticoids treatment in 2–3 weeks, until the goal of the normalization of inflammatory markers (CRP, ferritin, IL-6), troponin, PRO-BNP, D-Dimer, AST, ALT, gamma-GT, pancreatic amylase, lipase, lymphocyte count. Treatment with low dose of acetylsalicylic acid (ASA) (3–5 mg/Kg/day) was continued for 8 weeks, or until the resolution of CAL.

The cardiac MRI was performed during the hospitalization or after the discharge (the interval time occurred between the diagnosis and cardiac MRI was 0.3-7 months). The cardiac MRI was performed with a 1,5 Tesla scanner (GE Signa Explorer). The cardiac MRI study was not performed during the acute phase of MIS-C in some children, because they were clinically unstable and needed sedation or general anesthesia.

The protocol included, before intravenous contrast media infusion, retrospective ECG-Gated fiesta cine sequences (short axis, 4, 3 and 2 chamber views), sequences for oedema, and hyperemia T2 -short tau inversion recovery (Stir) (repetition time of 1689 ms, echo time of 55.10 ms).

Myocardial oedema was evaluated following the Lake Louise criteria. Because the lack of consistent reference of normal value in native T1 mapping and T2 relaxation time in the pediatric age, myocardial edema was defined by increased signal intensity on T2-weighted imaging and myocardial damage by non-ischemic patterns late gadolinium enhancement.

The study to evaluate myocyte necrosis and fibrosis considered late gadolinium-enhanced 2D inversion recovery sequences performed 6 min later intravenous contrast medium infusion (0,2 mmol/kg body weight gadoterate meglumine, Dotarem, Guerbet, France).

### Statistical analysis

All ordinal data were expressed as numbers and percentages. Descriptive statistics included mean, standard deviation (SD), median. Significance level was set at 95% (*p*-value < 0.05). For qualitative data, comparisons were performed using 2 × 2 contingency tables, applying *χ*^2^ test. We used the two-tailed Student’s t test to evaluate any statistically significant differences between the variables considered in the two groups of patients. We verified the results of the Student’s t-test with the two-tailed Mann-Whitney U test.

## Results

ECG was reported as abnormal in ten patients (50%) (5 males; 5 females), during the acute phase of MIS-C, of which four patients (20%) with isolated abnormalities of the repolarization phase; one patient with incomplete right bundle branch block associated with abnormalities of the repolarization phase; one patient with a Brugada pattern. Four patients (20%) showed tachycardia in the acute phase, followed by sinus bradycardia, associated with abnormalities of the repolarization phase in two of them. Eight patients with abnormalities of ECG, showed also valvular insufficiency.

Serial controls by ECG were performed during the follow-up, showing the resolution of ECG abnormalities in all the patients during the subacute phase of the disease, with the exclusion of the patient with Brugada pattern.

Transthoracic echocardiography demonstrated signs of cardiac involvement in all the patients, with different pattern. Fourteen patients showed valvular insufficiency: eight showed Mitral insufficiency; two showed Mitral and Aortic insufficiency; two showed Mitral and Tricuspid insufficiency; one showed Tricuspid insufficiency; one developed Mitral, Tricuspid and Aortic insufficiency.

Eight patients showed pericarditis; 4/8 patients (50%) with pericarditis showed an association with pleuritis and/or ascites: three with pleurites and ascites; one with ascites. 100% of them had valvular insufficiency. Contrarywise, between the 4/8 patients with pericarditis without pleuritis and/or ascites, the incidence of valvular insufficiency was lower (50%) (Table 2).

**Table 2:** Cardiological signs and symptoms of the patients

Patient (age in years)	ECG abnormalities	Pleuritis	Ascitis	Mesenteric adenitis	Pericarditis	Valvular insufficiency	CAL	MAS	Shock	Cardio-MRI (1) (months after the MIS-C)
**1 (12.7 years) M**	**+**	**-**	**+**	**+**	**-**	**+ (M)**	**-**	**-**	**-**	Normal (0.7)
**2 (6 years) F**	**+**	**-**	**-**	**-**	**-**	**+ (M)**	**-**	**-**	**-**	Normal (2)
**3 (4 years) F**	**-**	**-**	**-**	**+**	**+**	**-**	**-**	**-**	**-**	Normal (2)
**4 (2.8) F**	**-**	**-**	**+**	**-**	**-**	**-**	**-**	**+**	**-**	Normal (3)
**5 (2 years) M**	**-**	**-**	**-**	**-**	**-**	**-**	**+**	**-**	**-**	Normal (1)
**6 (16 years) M**	**+**	**+**	**+**	**-**	**+**	**+ (M)**	**-**	**-**	**+**	Oedema and delayed myocardial enhancement basal and inferior-basal wall; acute myocarditis (0.3)
**7 (2 years) F**	**+**	**-**	**-**	**+**	**-**	**-**	**-**	**-**	**-**	Normal (1)
**8 (11) M**	**+**	**-**	**+**	**+**	**-**	**+ (T)**	**+**	**-**	**-**	Normal (4)
**9 (5.4) F**	**-**	**-**	**+**	**+**	**+**	**+ (M)**	**-**	**+**	**-**	Normal (1)
**10 (9.4) M**	**+**	**+**	**-**	**+**	**-**	**+ (M)**	**-**	**-**	**-**	Normal (1)
**11 (10.7 years) F**	**-**	**-**	**-**	**-**	**+**	**-**	**-**	**-**	**-**	Mild pericardial effusion (7)
**12 (3.5 years) M**	**+**	**+**	**+**	**-**	**-**	**+ (M; T)**	**+**	**+**	**+**	Left ventricle apex focal edema (0.7)
**13 (11.7) F**	**+**	**-**	**+**	**+**	**-**	**+ (M)**	**-**			Normal (1)
**14 (2) M**	**-**	**-**	**-**	**-**	**-**	**+ (A; M)**	**-**	**-**	**-**	Apical septum spot edema (0.33)
**15 (12.7 years) M**	**-**	**-**	**-**	**-**	**+**	**+ (M)**	**-**	**-**	**-**	Pericardial effusion; myocardial delayed enhancement. Inferobasal wall Left ventricle fibrosis (1.3)
**16 (9.9 years) F**	**+**	**+**	**+**	**-**	**+**	**+ (A; M; T)**	**-**	**-**	**+**	Normal (0.33)
**17 (14) F**	**-**	**-**	**-**	**-**	**-**	**+ (A; M)**	**+**	**-**	**-**	Mild aortic insufficiency (3)
**18 (11) M**	**-**	**+**	**+**	**+**	**+**	**+ (M)**	**-**	**-**	**-**	Normal (2)
**19 (3) F**	**+**	**-**	**-**	**+**	**-**	**-**	**-**	**-**	**-**	Mild delayed enhancement for fibrosis of the middle part of the interventricular septum (1)
**20 (10.4) F**	**-**	**-**	**-**	**-**	**+**	**+ (M; T)**	**-**	**-**	**-**	Mild pericardial effusion (0.33)

8/14 patients (57%) with valvular insufficiency had in association ascites and/or pleuritis; 6/14 patients (43%) with valvular insufficiency had in association pericarditis. 3/14 patients (21%) with valvular insufficiency showed CAL (Table 2).

Four patients showed CAL without aneurisms. 100% of the patients with CAL had rash, cheilitis and/or pharyngitis, lymphadenopathy, vomiting and/or abdominal pain. 3/4 patients (75%) with CAL had in association valvular insufficiency of two valves in two children, valvular insufficiency of one valve in one child.

8/14 patients (57%) with valvular insufficiency had arrythmia; 6/14 patients (43%) with valvular insufficiency did not show ECG abnormalities.

All patients underwent cardiac MRI 0.3-7 months after the acute stage of MIS-C. The imaging study revealed that eight children (40%) (4 M; 4 F) (Table 1), showed pathological cardiac MRI: two children showed pericardial effusion; however, pericardial effusion resolved during the follow-up in both. One patient showed a persistent aortic insufficiency. Two children showed myocardial oedema. Three patients showed delayed enhancement: one child showed oedema associated with delayed enhancement for acute myocarditis; two children showed delayed enhancement associated with myocardial fibrosis. Delayed enhancement persisted in all three patients. Between those patients with pathological cardiac MRI, 4/5 patients with myocardial oedema and/or fibrosis repeated the cardio MRI after 5.6-9 months. Only one patient with myocardial oedema had a low compliance and did not repeat the cardiac MRI but was followed by echocardiography.

Cardiac MRI repeated during the follow-up documented a significant reduction of the extension of fibrosis and the disappearance of the oedema.

Among the 12/20 patients with normal cardiac MRI, T2-Stir sequences demonstrated neither myocardial oedema nor hyperemia nor fibrosis in 12/20 patients (60%).

The cardiac MRI with normal results were performed 0.3-4 months after the acute phase of the disease. Furthermore, among the twelve patients with normal cardiac MRI, two (17%) had CAL, associated with valvular insufficiency of one valve in only one child.

Among the 8/20 patients with pathological cardiac MRI, two (25%) had CAL and valvular insufficiency of two valves. These data highlight the higher risk of CAL in patients with other cardiac pathological findings.

Four patients were treated with anakinra at a variable dosage (2–8 mg/Kg/day) depending on the severity of the cardiac involvement.

Between those, two patients showed a normal cardiac MRI after 0.33 and 2 months respectively.

Two patients showed pathological signs documented by cardiac MRI: in one patient, a pathological cardiac MRI documented left ventricle apex focal oedema 0.7 months after the acute stage of the disease.

The follow-up showed the resolution of cardiac lesions. In one patient cardiac MRI showed pericardial effusion, associated with myocardial delayed enhancement, inferobasal wall left ventricle fibrosis, documented 0.33 months after the acute stage of the disease. Cardiac MRI, performed 6 months later, documented a significant reduction of fibrosis. The patient is still in follow-up, with a late normalization of the cardiologic lesions.

Not all patients with pathological findings on MRI were able to have a cardiac MRI during the follow-up, because they needed sedation and, in some cases, it was not allowed by their parents. However, they were followed by echocardiography and ECG, that documented the resolution of pathological conditions, as CAL, valvular insufficiency, pericardial effusion. The patients with fibrosis were followed by cardiac MRI, except one, who had a low familial compliance. The patient had acute myocarditis with shock, CAL and Mitral and Tricuspid insufficiency. He was followed by echography, that showed the resolution of myocardial insufficiency with restored ejection fraction, and the regression of CAL and of valvular insufficiency after anakinra treatment.

Haematological parameters were correlated with cardiac lesions, with the relieve of pleuritis or ascites, and with clinical signs and symptoms at the diagnosis. Nonetheless, we did not find any statistically significant correlation. Patients with normal cardiac MRI had higher levels of ferritin, triglycerides, gamma-GT. Patients with pathological cardiac MRI had higher levels of procalcitonin (23.30 ± 43.41 vs. 3.69 ± 3.22 µg/l) and IL-6 (285.5 ± 502.7 vs. 1427.3 ± 3489.9 pg/ml) than patients with normal cardiac MRI (Supplementary Table 1). However, these parameters did not reach a statistically significant difference between the two groups of patients for the wide range of the SDS calculated for these parameters. These data support the evidence of the high inflammatory status both in patients with and without pathological cardiac MRI.

## Discussion

Cardiac MRI imaging studies on myocardial injury in children affected by MIS-C are few in the literature and limited to cohorts of a small number of patients [9–12, 16–18].

Transient myocardial dysfunction or severe myocarditis with pump failure, are described in children with MIS-C. The full recovery occurs in most of them. Coronary lesions, valve regurgitation and pericardial effusion are associated signs of cardiac involvement.

An Italian study on 17 patients with MIS-C documented CAL in 26.1% and late gadolinium enhancement in 35.2% of patients [9].

Some patients evolve into the full recovery of the cardiac involvement [8]; in some patients, cardiac damage can persist during the follow-up.

Arslan S.Y. et al., in a study of 34 patients, demonstrated that patients with MIS-C had a high rate of cardiac involvement, particularly pericardial effusion and left ventricular dysfunction showed by cardiac MRI, performed at 2–6 months after the diagnosis. They suggest employing cardiac MRI in MIS-C patients in the late period, to document persistent cardiac involvement also in patients with normal echocardiography in the acute phase of the disease [10].

The pathogenesis of heart involvement is still object of study; however, the role of cytokines storm is well documented [8, 19]. Proinflammatory cytokines are implicated in the genesis of cardiac damage. These data are the pathogenetic rationale for therapeutic choices. In fact, both IVIG and glucocorticoids inhibit the immune-mediated response. Furthermore, anakinra plays a role in the target therapy of these patients, blocking the action of IL-1 alpha and beta, antagonizing IL-1 receptor [6, 20–22].

Treatment with anakinra showed a significant and persistent efficacy in multifactorial autoinflammatory diseases, as systemic Juvenile Idiopathic Arthritis (sJIA). Efficacy of anakinra did not show any significant differences between sJIA and AOSD patients, and between patients treated in monotherapy compared to patients treated with conventional disease-modifying antirheumatic drugs (cDMARDs) [23].

Following the success of canakinumab in the treatment of autoinflammatory diseases as sJIA [24, 25] and monogenic autoinflammatory diseases [26–28], and the low efficacy in recurrent pericarditis, the anti-IL-1 beta blockade is not considered the target treatment in MIS-C. Therefore, the treatment of choice, in patients with a severe clinical presentation and/or non-responders to IVIG and glucocorticoids, remains anakinra as documented in KD [29].

Four patients of our case series were treated with anakinra at a variable dosage depending on the severity of the cardiac involvement. 3/4 patients showed the resolution of cardiologic involvement in the mid-term follow-up. One patient showed a late resolution of cardiologic lesions. These results confirm the efficacy of anakinra in the treatment of severe MIS-C patients.

A multicenter retrospective cohort study demonstrated that the early treatment with anakinra was safe and was associated with a lower incidence of persistent heart disease at the end of follow-up. The study reports that early treatment with anakinra is effective in patients with a high risk of a severe disease outcome [22].

Furthermore, patients with MIS-C need a strict follow-up, to demonstrate the resolution of cardiac damage. They need to repeat ECG to document the normalization of the heart rhythm. A recent meta-analysis relieved ECG anomalies in 5.3% of children with MIS-C [30]. In our case series arrythmia was in 50% of patients in the acute stage of the disease.

Echocardiography is required to monitor children with a fast and frequently replicable imaging. However, to deeply analyze these patients, we need cardiac MRI, which can study the tissue characterization, can evidence oedema, late contrast enhancement and fibrosis in the acute stage of the disease. Indeed, cardiac MRI contribute to guide the therapeutic approach, especially in patients with myocadiac oedema and/or fibrosis, because ECG and echocardiography are unable to tissue characterization.

Cardiac MRI defines the presence or absence of myocardial inflammation; allows to accurately evaluate the extent of inflammation of the myocardium and pericardium, any myocardial fibrosis, and other associated cardiac anomalies; documents the complete or partial resolution of these sequelae, not detectable by echocardiography, during the follow-up. These parameters are essential for a target therapy and guide the clinician in choosing to intensify the therapeutic strategy (dose of steroid, inclusion of anakinra in therapy). Furthermore, they are fundamental data for the tapering of glucocorticoid and/or anakinra therapy. Therefore, MRI helps to define responder and non-responder patients to first level therapy, directing towards an intensification of care.

Our patients with pericarditis had an increased risk of other systemic and/or cardiac involvement. Furthermore, the risk of valvular insufficiency was higher (100% of all the children) when serositis of one or more districts was associated with pericarditis. Additionally, CAL were associated with valvular insufficiency in 75% of children, and arrythmia was associated with valvular insufficiency in 57% of patients.

All the patients with CAL showed rash, cheilitis and/or pharyngitis, lymphadenopathy, manifesting many clinical signs common to MIS-C and KD, expression of a high inflammatory degree documented in these patients.

In our population we observed that the involvement of other districts increases the risk of further cardiac lesions. These data support the role of proinflammatory cytokines in the pathogenesis of MIS-C [8, 19].

Furthermore, CAL are the expression of a specific organ vasculitis, with a possible unfavorable course, and CAL, as expression of vasculitis, are described also in infectious diseases [31] or in KD triggered by infectious agents [29, 32]. Vasculitis associated with SARS-CoV-2 infection was described in children, both in the acute phase of COVID-19 or after two or more weeks from SARS-CoV-2 exposure [33], and the pathogenesis of vasculitis may overlap with that of MIS-C. It is probably the result of the exaggerated immune response triggered by the virus. Besides, vasculitis is mediated by several steps of endothelial injury and vascular lumen thrombosis. The downregulation of angiotensin‐converting enzyme 2 by SARS–CoV‐2 binding affects an increase in angiotensin II activity, contributing to vasoconstriction. Moreover, the viral direct cytopathic effect may induce endothelitis with the endothelial barrier loss, unmasking the basement membrane to platelets. Hence, the thrombosis cascade needs anti-coagulant treatment and a strict follow-up [33].

Most cardiac MRI examinations of our patients did not document the presence of myocardial edema, probably because the study was performed during the subacute stage of the disease. However, the lower incidence of pathological images on cardiac MRI in our series, compared to that reported in other studies [10], can depend on the early and proper diagnosis and treatment of our patients.

Although all the children with MIS-C included in the study had cardiac compromise in the acute phase of the disease, residual myocardial damage during the follow-up appears minimal and limited to a few cases. In fact, cardiac involvement can be relieved during the acute phase of MIS-C, but cardiac compromise generally does not lead to persistent damage during the follow-up.

Our study evaluated the prevalence and the low persistence of residual cardiovascular pathology in determining long-term prognosis. However, late gadolinium contrast enhancement can disappear at the follow-up cardiac MRI study, but in some cases this data depends on the resolution of edema or scare shrinkage below the MRI resolution level [34].

In a recent study, a high incidence of persistent cardiac sequelae was observed. The authors demonstrated a residual subclinical cardiovascular pathology in many patients with MIS-C [16].

For this reason, we emphasize the possible need to intensify care during follow-up, to manage cardiologic sequelae and to prevent further medium and long-term complications.

## Conclusions

Cardiac MRI is an excellent, standardized, noninvasive diagnostic examination for the diagnosis and follow-up of heart diseases, focusing some aspects of myocardial damages: oedema, inflammation, pericardial effusion, contractile scar impairment and necrosis. It can identify patients at risk to develop arrhythmias and unfavorable outcome.

Even in patients with normal echocardiography in the acute phase of MIS-C, cardiac MRI should be performed during the follow-up. In fact, all patients with a diagnosis of MIS-C require cardiac follow-up, regardless of the severity of the disease at the diagnosis. Cardiac MRI is a valuable tool to evaluate the degree of cardiac involvement and must be included in the follow-up protocol of these patients.

Indeed, ECG is needed to study rhythm abnormalities and to evaluate the resolution of eventual arrythmia, often observed in the acute stage of the disease. Echocardiography is needed to follow children, with a fast and frequently replicable imaging. Nonetheless, ECG and echocardiography are unable to study tissue characterization. However, our goal is to arrive to the heart of the problem. We need cardiac MRI, which can evidence oedema, late contrast enhancement and fibrosis. Therefore, we underline the importance of carrying out MRI even in the late stages of the disease, to identify the persistence of cardiac damage, even in patients who had normal echocardiogram in the acute phase of MIS-C.

### Electronic supplementary material

Below is the link to the electronic supplementary material.

Supplementary Material 1

Supplementary Material 2

Supplementary Material 3

## Data Availability

The datasets used and/or analysed during the current study are available from the corresponding author on reasonable request.

## Abbreviations

MIS-C: multisystem inflammatory syndrome in children
CAL: coronary artery lesions
KD: Kawasaki disease
MRI: magnetic resonance
CDC: Centers for Disease Control and Prevention
CRP: C-reactive protein
ESR: erythrocyte sedimentation rate
IVIG: intravenous immunoglobulins
ASA: acetylsalicylic acid
SARS-CoV-2: severe acute respiratory syndrome coronavirus 2
IL-6: interleukin 6
MAS: Macrophage Activation Syndrome
WHO: World Health Organization
