# Supplementary material for: Cardiovascular involvement in children with COVID-19 temporally related multisystem inflammatory syndrome (MIS-C): can cardiac magnetic resonance arrive to the heart of the problem?
Source: Ital J Pediatr. 2024 May 3;50:91. doi: 10.1186/s13052-024-01658-1 (PMC11071142; doi:10.1186/s13052-024-01658-1)
Supplement: Supplementary file 2 — Supplementary Material 2 [file 13052_2024_1658_MOESM2_ESM.docx]

Supplementary Table 1: M ± SDS of laboratory parameters of the patients.

| Patients | Hb g/dl | WBC/µL | Neutrophils/lymphocytes/ µL | Platelets/µL | CRP (n.v. <0.5 mg/dl) | Procalcitonin (µg/l) | Protein/albumin (g/dl) | Creatinine (mg/dl) | Na (mmol/l) | AST /ALT (UI/l) | Gamma GT (UI/l) | LDH (UI/l) | Troponine (ng/l) | PRO-BNP (pg/ml) | D- Dimer | Fibrinogen (mg/dl) | Ferritin (ng/ml) | Triglyceride (mg/dl) | IL-6 (pg/ml) |
| --- | --- | --- | --- | --- | --- | --- | --- | --- | --- | --- | --- | --- | --- | --- | --- | --- | --- | --- | --- |
| total | 11.55 ± 1.32 | 11018 ± 6574 | 7.4 ± 6.8 | 215400 ± 106008 | 16.85 ± 15.12 | 9.46 ± 25.32 | 6.50 ± 0.65/3.6 ± 0.15 | 0.60 ± 0.32 | 133 ± 3.9 | 46.92 ± 25.16/42.65 ± 34.1 | 37 ± 45.2 | 308.3 ± 119.1 | 102.7 ± 203.9 | 4008.9 ± 7177.7 | 207.9 ± 562.7 | 821.9 ± 965.2 | 978.7 ± 1682.5 | 238.4 ± 173.3 | 970.5 ± 2778.7 |
| Pathological cMRI | 11.49 ± 1.42 | 9791 ± 3258 | 6.1 ± 6.4 | 221488 ± 50320 | 19.72 ± 18.64 | 23.30 ± 43.41 | 6.54 ± 0.72/3.68 ± 0.38 | 0.69 ± 0.36 | 134 ± 2 | 48.13 ± 47.75/48.13 ± 47.75 | 19.8 ± 11.7 | 290.1 ± 115.1 | 54 ± 115.3 | 3354.3 ± 3816.9 | 193.7 ± 422.8 | 488.6 ± 252.9 | 1630.1 ± 2397.2 | 163.8 ± 109.6 | 285.5 ± 502.7 |
| Normal cMRI | 11.59 ± 1.25 | 11836 ± 7955 | 6.9 ± 6.7 | 211341 ± 130385 | 14.76 ± 11.48 | 3.69 ± 3.22 | 6.47 ± 0.59/3.55 ± 0.58 | 0.54 ± 0.26 | 132.1 ± 4.6 | 47.4 ± 45.95/49.5 ± 38.9 | 47.9 ± 54.3 | 321 ± 120 | 179 ± 276.5 | 4581.7 ± 9118.5 | 216.4 ± 631.9 | 973.4 ± 119.2 | 490.2 ± 294.7 | 291.7 ± 190 | 1427.3 ± 3489.9 |
