# Supplementary material for: Cardiovascular involvement in children with COVID-19 temporally related multisystem inflammatory syndrome (MIS-C): can cardiac magnetic resonance arrive to the heart of the problem?
Source: Ital J Pediatr. 2024 May 3;50:91. doi: 10.1186/s13052-024-01658-1 (PMC11071142; doi:10.1186/s13052-024-01658-1)
Supplement: Supplementary file 1 — Supplementary Material 1 [file 13052_2024_1658_MOESM1_ESM.docx]

Dear Reviewers,

we include the point-by-point response, as required.

Best regards,

the authors

Reviewer reports:

Reviewer #1: The article addresses the topic of MIS-C (Multisystem Inflammatory Syndrome in Children), a hyperinflammatory syndrome that occurs after exposure to SARS-CoV-2 infection in patients under the age of 16. The author describes the diagnostic criteria, common symptoms, and cardiovascular complications associated with MIS-C. The article is based on a retrospective study conducted on 20 children with cardiac involvement secondary to MIS-C.
Strengths of the article:
Clear context: The author provides a clear overview of the context, introducing the MIS-C theme and explaining its relationship with SARS-CoV-2 infection.
Detailed methodology: The article provides details on the study methodology, including patient inclusion criteria, administered treatment, and imaging techniques used (ECG, echocardiogram, cardiac magnetic resonance).
Presentation of results: The study results are presented clearly and in detail, with a discussion on the prevalence of cardiac complications and the effectiveness of treatment.
Potential weaknesses of the article:
Study limitations: The author mentions that the study is retrospective and conducted in a single center, which could limit the generalizability of the results.
Details on the study population: Some details about the study population, such as the average follow-up duration and specific patient characteristics, could be further elaborated.

We added a table with patients data, as required.

Discussion on comparison with other research: While the author references previous studies, a more in-depth discussion on comparing the results with existing literature could enhance the robustness of the conclusions.

Thank you for the support. We discussed the results of other study, as suggested.

Reviewer #2: I would like to point out first of all that the title of the article suggests a question to which the authors don't give an answer either in the discussion or in the conclusions.

We added the answer in the discussion and in the conclusions, as suggested.

Results: The presentation is difficult to follow and only table 1 allows for a clear understanding of the evaluation methods of the 20 cases.
I would propose to begin the results by reporting that all patients underwent ECG and echocardiography and only subsequently reporting the cases also examined with MRI.

All the patients were examined with MRI. We highlighted this element in the methods.

Some questions :
- How many children with pathological MRI repeated the exam after a few months?

4/5 patients with myocardial oedema and/or fibrosis, repeated cardio MRI after 5.6-9 months. One patient with myocardial oedema had a low compliance and did not repeat cardiac MRI but was followed by echocardiography. We added this information in the results, as required by the Revisor.

- Is there a statistically significant difference in the parameters reported in Table 2, in particular procalcitonin and interleukin, between patients with pathological MRI and normal MRI?
-Otherwise the table could be replaced with a short summary text.

All the parameters did not reach a statistically significant difference, however the medium levels of IL-6 and procalcitonin were higher in the group of patients with pathological MRI. Furthermore, the wide range of the SDS did not permit to reach a statistically significant difference. We added these data to the “Results”- We maintained the table (now called “Table 3”) as supplementary material for specific consultation.

Discussion: contains a lot of informations concerning the therapy and the clinical course of patients affected by MIS-C but doesn't address the question that the title of the article proposes.

Thank you for your consideration. We discussed this issue in the discussions.

This issue is only partially discussed in the conclusions.

Thank you for your consideration. We discussed this issue also in the conclusions.

I would expect the authors to argue about some questions : based on this experience, what are the potentials of MRI in the management of children affected by MIS-C?

Cardiac MRI defines the presence or absence of myocardial inflammation; documents the persistence of myocardial involvement not detectable by echocardiography; allows you to precisely evaluate the extent of inflammation of the myocardium and pericardium, any myocardial fibrosis, and other associated cardiac anomalies. These parameters are essential for choosing the therapy and possibly intensifying the therapeutic strategy (dose of steroid, inclusion of anakinra in therapy). Furthermore, they are fundamental data for choosing the tapering of glucocorticoid and/or anakinra therapy. MRI therefore helps to define responder and non-responder patients to first level therapy, directing towards an intensification of care. We reported these comments in the discussion.

Do MRI really change the therapeutic approach or, as it seems, are ECG and echocardiography adequate for the management of MIS-C?

MRI contribute to guide the therapeutic approach, especially in patients with myocadiac oedema and/or fibrosis, because ECG and echocardiography are unable to tissue characterization. We added these comments in the discussion.

Also considering the unstable conditions of many patients, MRI is a test that is difficult to perform routinely?

Yes, as defined in the text.

Is it more useful for evaluating any cardiac outcomes?

It is useful to define both sequel and acute damage.

If the authors agree with these observations, also the abstract should be profoundly modified and in any case should be shortened.

We changed and shortened the abstract, as suggested.
