# Supplementary material for: Cardiovascular involvement in children with COVID-19 temporally related multisystem inflammatory syndrome (MIS-C): can cardiac magnetic resonance arrive to the heart of the problem?
Source: Ital J Pediatr. 2024 May 3;50:91. doi: 10.1186/s13052-024-01658-1 (PMC11071142; doi:10.1186/s13052-024-01658-1)
Supplement: Supplementary file 3 — Supplementary Material 3 [file 13052_2024_1658_MOESM3_ESM.docx]

Dear Editor,

we submit the paper on cardio MRI study of patients with MIS-C. This monocentric study evidenced a good cardiologic prognosis of children with cardiac involvement in the acute stage of the disease. A prompt diagnosis and the efficacy of treatment allowed this goal. We followed our patients with echocardiography and ECG and with cardio MRI, to follow and evidence possible cardiologic sequelae.

We think that the paper highlights the role of cardio MRI in the follow-up of children with MIS-C.

Authors have no potential competing interests.

All authors have approved the manuscript for submission.

The content of the manuscript has not been published or submitted for publication elsewhere.

Best regards,

The authors
